# The Potential Role of Ferroptosis in Neonatal Brain Injury

**DOI:** 10.3389/fnins.2019.00115

**Published:** 2019-02-14

**Authors:** Yanan Wu, Juan Song, Yafeng Wang, Xiaoyang Wang, Carsten Culmsee, Changlian Zhu

**Affiliations:** ^1^Henan Key Laboratory of Child Brain Injury, Third Affiliated Hospital of Zhengzhou University, Zhengzhou, China; ^2^Institute of Neuroscience and Physiology, Sahlgrenska Academy, University of Gothenburg, Gothenburg, Sweden; ^3^Institute of Pharmacology and Clinical Pharmacy, University of Marburg, Marburg, Germany; ^4^Center for Mind, Brain and Behavior, University of Marburg, Marburg, Germany; ^5^Center for Brain Repair and Rehabilitation, Institute of Neuroscience and Physiology, Sahlgrenska Academy, University of Gothenburg, Gothenburg, Sweden

**Keywords:** hypoxic ischemic brain injury, intraventricular hemorrhage, cell death, iron toxicity, lipid peroxidation, neonate, reactive oxygen species, lipid peroxidation

## Abstract

Ferroptosis is an iron-dependent form of cell death that is characterized by early lipid peroxidation and different from other forms of regulated cell death in terms of its genetic components, specific morphological features, and biochemical mechanisms. Different initiation pathways of ferroptosis have been reported, including inhibition of system X_c_^-^, inactivation of glutathione-dependent peroxidase 4, and reduced glutathione levels, all of which ultimately promote the production of reactive oxygen species, particularly through enhanced lipid peroxidation. Although ferroptosis was first described in cancer cells, emerging evidence now links mechanisms of ferroptosis to many different diseases, including cerebral ischemia and brain hemorrhage. For example, neonatal brain injury is an important cause of developmental impairment and of permanent neurological deficits, and several types of cell death, including iron-dependent pathways, have been detected in the process of neonatal brain damage. Iron chelators and erythropoietin have both shown neuroprotective effects against neonatal brain injury. Here, we have summarized the potential relation between ferroptosis and neonatal brain injury, and according therapeutic intervention strategies.

## Introduction

Neonatal brain injury is an important cause of developmental impairment and of permanent neurological deficits such as cerebral palsy in children. Among many etiological factors, hypoxic–ischemic encephalopathy in term infants and intraventricular/periventricular hemorrhage in preterm infants are the most common causes of neonatal brain damage (Bennet et al., 2012; Shankaran et al., 2014). The progression of brain injury depends on the balance between persistent injury and the repair response, which can be modulated by therapeutic intervention (Kaandorp et al., 2012; Azzopardi et al., 2016; Song et al., 2016). Emerging evidence indicates that there is great potential for improving the treatment of acute brain injury in these children as well as opportunities for more effective regenerative treatment of these patients (Juul and Ferriero, 2014; O’Gorman et al., 2015; Tagin et al., 2015). Today, cooling the body is the only established method of treating newborns after asphyxia, but the protective effects are limited and only moderately injured children benefit from this treatment (Azzopardi et al., 2014). It is imperative, therefore, that we continue our efforts to identify the mechanisms of injury and repair in the developing brain and to identify new therapeutic strategies. An improved understanding of the mechanisms of brain injury is needed in order to develop strategies for the next generation of treatments for brain injuries in both term and preterm infants.

Neuronal cell death after an insult takes several different forms, but the underlying mechanisms of neuronal death share common features and signaling pathways. However, the cell death mechanisms in the developing brain have been shown to be quite different compared to those in the adult brain (Zhu et al., 2005; Wang et al., 2009). Neuronal cell death can be classified into accidental and regulated forms. Accidental cell death is induced by severe insults that cause immediate cellular demise, usually necrosis, and these cells cannot be rescued (Galluzzi et al., 2018). In contrast, regulated cell death has been differentiated into many types defined by morphological and/or biochemical features, including apoptosis, necroptosis, autophagy, pyroptosis, eryptosis, and more recently also ferroptosis (Galluzzi et al., 2018). Ferroptosis was first described in RAS mutant cancer cells in 2012 and has been defined as an iron and lipid peroxidation-dependent form of cell death that is genetically, biochemically, morphologically, and mechanistically distinct from other types of cell death (Dixon et al., 2012; Stockwell et al., 2017).

Studies elucidating the mechanisms of different ferroptosis inducers have found that system X_c_^-^ and glutathione-dependent peroxidase 4 (GPX4) inhibition or depletion can trigger ferroptosis through reduced glutathione (GSH) levels and subsequent accumulation of reactive oxygen species (ROS), especially through enhanced lipid peroxidation (Seiler et al., 2008; Stockwell et al., 2017; Seibt et al., 2018). Ferroptosis has been found not only in cancer cells, but also in dying neurons in model systems of neurological disorders (Weiland et al., 2018; Wu et al., 2018). Prior to the discovery of ferroptosis, the iron chelator deferoxamine (DFO) had been shown to have great potential in protecting brain cells from death (Lee et al., 2011; Masaldan et al., 2018); however, the mechanism behind this activity remained unknown, but recent studies on ferroptosis might provide an explanation (Dixon et al., 2012; Xie Y. et al., 2016; Morris et al., 2018). Programmed cell death is important for normal development, and such activity is more pronounced in neonates than in adults (Wang et al., 2009). Furthermore, metabolic iron imbalance is common and antioxidant capacity is low in neonates; and this suggests that ferroptosis might be important in neonates under pathological conditions. However, there are as yet no reports or reviews linking ferroptosis and neonatal brain injury. Here, we summarize recent advances in our understanding of ferroptosis, and we discuss the potential relationship between ferroptosis and neonatal brain injury.

## Definition and Discovery of Ferroptosis

Ferroptosis is a non-apoptotic form of cell death that depends on cellular iron and ROS (Dixon et al., 2012), and ferroptosis inducers had been applied before the mechanisms of ferroptosis were first proposed. Erastin, a synthetic compound, was discovered in Dolma et al. (2003). They found that erastin could cause non-apoptotic cell death in cells that expressed an engineered mutant Ras protein, but not in their wild-type counterparts. Later in 2008, they used high- throughput screening of small-molecule libraries to identify two Ras-selective lethal small molecules (RSL3 and RSL5) that induce non-apoptotic and iron-dependent oxidative cell death, and they found that the cell death could be inhibited by the iron chelator desferrioxamine mesylate and by the antioxidant vitamin E (Yang and Stockwell, 2008). The mode of cell death induced by RSL3 was found to be non-apoptotic because these cells showed no apoptotic hallmarks and because such cell death still occurred in cells where the factors of the core apoptosis machinery – i.e., caspases, BAX, and BAK – were suppressed (Yang and Stockwell, 2008; Wolpaw et al., 2011; Dixon et al., 2012). In 2012, Dr. Stockwell coined the term “ferroptosis” to describe this newly discovered form of cell death (Dixon et al., 2012). The morphological features of ferroptosis include an intact cell membrane without ruptures or blebs, normal nucleus size, and a lack of chromatin condensation, but with a shrinking mitochondrial membrane that shows increased membrane density and reduced or complete absence of mitochondrial cristae as well as outer mitochondrial membrane rupture (Dixon et al., 2012; Xie Y. et al., 2016; Doll and Conrad, 2017). Ferroptosis inducers can be roughly divided in two classes, one is represented by system X_c_^-^ inhibitors such as erastin and sulfasalazine and the other acts through GPX4 inhibition such as RSL3 (Stockwell et al., 2017). Accompanied with the discovery of inhibitors of ferroptosis, the veil of ferroptosis has gradually been lifted.

## Metabolic Pathways and Molecular Mechanisms in Ferroptosis

### Inhibition of System X_c_^-^ Triggers Ferroptosis

**FIGURE 1:**
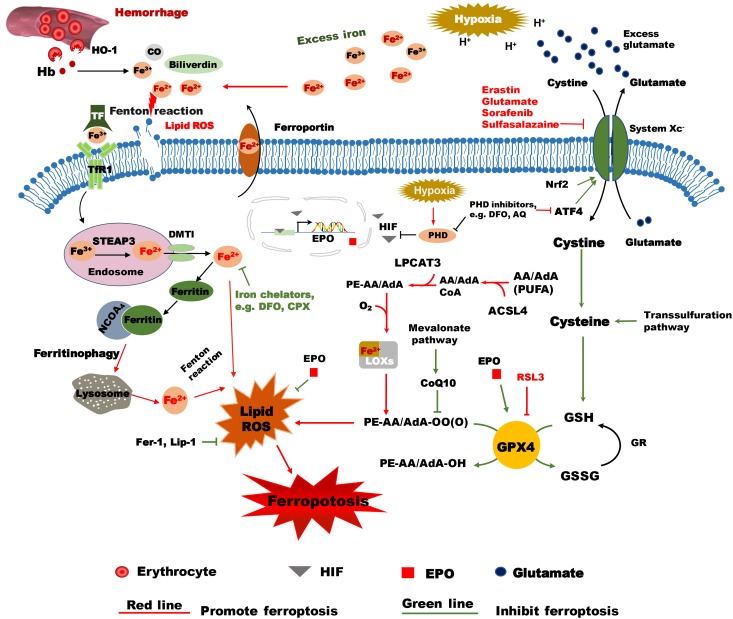
Hypothetical mechanisms of hypoxia ischemia and intracranial hemorrhage-induced ferroptosis in the immature brain. Following intracranial hemorrhage, the lysed erythrocytes release Hb, which produces a degradation product, heme, that is then degraded by HO-1 into carbon monoxide CO, biliverdin, and free iron. Excess Fe^2+^, the reactive form of iron, will cause membrane lipid damage and ferroptosis. The selective ferroptosis inhibitors Fer-1 and Lip-1 inhibit lipid peroxidase activity and inhibit ferroptosis. Iron chelators such as DFO can reduce the level of unbound iron and inhibit the production of ROS and the occurrence of ferroptosis. EPO reduces the level of unbound iron by promoting erythropoiesis. The inactive form of iron (Fe^3+^) is recognized by TF and delivered into the cell by TfR1 and stored in endosomes, where Fe^3+^ is then converted into Fe^2+^ by STEAP3. Free iron (Fe^2+^) can be transported by DMT1 out of the endosome. Some of the free iron is stored in ferritin, some is transported out of the cell by ferroportin, and some might cause lipid ROS. Free iron can be released from ferritin degradation via the ferritinophagy pathway, which is mediated by NCOA_4_. These proteins work together to maintain the balance of iron as well as to control cell fate to some content. Erastin impedes cystine transport by inhibiting system Xc^-^, while Nrf acts in an opposing role by upregulating system Xc^-^ transcription. Once inside the cell, cystine is reduced to cysteine, and the level of cysteine can be supplemented by the trans-sulfuration pathway. Subsequently, cysteine is used for the biosynthesis of GSH. GPX4 uses two GSH molecules as electron donors to reduce phospholipid hydroperoxides (PL-OOH) to the corresponding alcohols leaving GSSG (oxidized GSH) as a byproduct. ACSL4 is required for activation of PUFAs, especially arachidonic acid (AA) and adrenic acid (AdA) to AA-CoA and AdA acyl co-A derivatives. These derivatives are esterified by LPCAT3 into AA-PE and AdA-PE, which then be catalyzed by the iron-containing enzyme lipoxygenase (LOX) to generate fatty acid hydroperoxides. After hypoxia-ischemia insult, the acidulated environment will cause the accumulation of excess iron and glutamate. Accumulated glutamate will inhibit system Xc^-^, which in turn will lead to insufficient levels of cellular cysteine and GSH production. As a result, GPX4 will be inactivated and lead to lipid peroxidation and ferroptosis. RSL3 is a ferroptosis activator that binds to and inactivates GPX4. During hypoxia insult, HIF is stabilized by PHDs and is translocated into the nucleus where it turns on transcription of EPO. EPO stimulates the activity of GPX4 and inhibits lipid peroxides and thus acts as an inhibitor of ferroptosis. PHD inhibitors like DFO and AQ not only inhibit PHDs, but also inhibit ATF4, which is a ferroptosis activator. AA/AdA, arachidonic acid/adrenic acid; ACSL4, acyl-CoA synthetase long chain family member 4; AIF, apoptosis inducing factor; AQ, adaptaquin; ATF4, activating transcription factor 4; CO, carbon monoxide; DFO, deferoxamine; DMT1, Divalent metal transporter 1; EPO, Erythropoietin; Fer-1, ferrostatin-1; GPX4, glutathione peroxidase 4; GR, glutathione reductase; GSSG, oxidized GSH; GSH, reduced glutathione; Hb, hemoglobin; HO-1, heme oxygenase 1; HIF, hypoxia-inducible factor; Lip-1, liproxstatin-1; LPCAT3, lysophosphatidylcholine acyltransferase 3; PE: phosphatidylethanolamine; PHDs, prolyl-hydroxylases; PL-PUFA (PE), polyunsaturated-fatty-acid-containing phospholipids; PL-PUFA(PE)-OOH, polyunsaturated-fatty-acid-containing- phospholipid hydroperoxides; PL-PUFA(PE)-OH; PUFA, polyunsaturated fatty acid; ROS, reactive oxygen species; RSL3, RAS-selective lethal 3; STEAP3, 6-transmembrane epithelial antigen of the prostate 3; TF, transferrin; TfR1, transferrin receptor 1; VDAC, Voltage-dependent anion-selective channel protein.

System X_c_^-^ is a heterodimeric cystine/glutamate antiporter composed of SLC7A11 (xCT) and SLC3A2, and it is responsible for maintaining redox homeostasis by importing cystine into the cell where it is further reduced to cysteine for synthesizing the major antioxidant GSH. Cysteine is the rate-limiting factor in cellular GSH biosynthesis because this amino acid is relatively rare in food. It has been shown that the cysteine-glutathione pathway is a pivotal upstream signaling regulator of ferroptosis (Dixon et al., 2014). Impairment of the system X_c_^-^-dependent antioxidant defense system results in oxidative injury and cell death. The small molecule erastin has been shown to inhibit system X_c_^-^ activity and lead to the accumulation of lipid ROS and thus to trigger ferroptosis (Dixon et al., 2012, 2014). Several other agents also induce ferroptosis through inhibiting system X_c_^-^, such as glutamate, sulfasalazine and sorafenib (Dixon et al., 2014) (Figure 1).

Glutamate is exchanged for cystine in a 1:1 ratio by system X_c_^-^, thus excess glutamate acts as an equivalent of erastin and can also induce ferroptosis, which perhaps provides new insight into explaining the mechanism of glutamate toxicity in the nervous system. Previous studies have shown that glutamate, at millimolar concentrations, can also block X_c_^-^ activity in fibroblasts and in a neuronal cell line, thereby exerting pathways of oxidative cell death that involve GSH depletion and lipid peroxidation (Tan et al., 1999; Tobaben et al., 2011; Neitemeier et al., 2017). This form of cell death in immortalized hippocampal neurons (HT22 cell line) was previously defined as oxytosis (Tan et al., 2001), and more recent data demonstrated similarities between ferroptosis and oxytosis at the level of X_c_^-^ inhibition, GSH depletion, and Gpx4 inhibition and in mitochondrial pathways of oxidative cell death in neurons that involve activation of the pro-apoptotic BID and the mitochondrial release of apoptosis inducing factor (AIF) (Neitemeier et al., 2017; Jelinek et al., 2018). The mitochondrial mechanisms merged the previously separated pathways of oxytosis and ferroptosis in neuronal cells, and this is supported by data demonstrating the involvement of AIF in cell death induced by genetic Gpx4 depletion (Seiler et al., 2008), and the particular role of mitochondrial AIF translocation to the nucleus of damaged neurons in models of neonatal hypoxia/ischemia (Zhu et al., 2003) and in transient focal cerebral ischemia (Plesnila et al., 2004; Culmsee et al., 2005). It is well established that glutamate toxicity plays an important role in neuronal cell death in neonatal brain injury (Zhu et al., 2010; Jantzie et al., 2015). The neurotoxicity of glutamate in differentiated neurons and brain tissue is dependent on excitotoxic (Tobaben et al., 2011) disruption of the Ca^2+^ homeostasis and oxidative stress mediated by oxidative iron species, and this can be inhibited by iron chelators or ferroptosis-specific inhibitors, including 12/15 LOX inhibition (Neitemeier et al., 2017). These findings demonstrate the close relationship between glutamate toxicity, system X_c_^-^ inhibition, lipid peroxidation, and ferroptosis.

In addition to glutamate, recent studies have found other biological molecules that regulate ferroptosis through system X_c_^-^. A recent study reported that beclin1 promotes ferroptosis by directly blocking system Xc^-^ activity. Mechanistically, beclin1 is phosphorylated by AMP activated protein kinase at Ser 90/93/96 and then interacts with SLC7A11, a core component of system Xc^-^, to prevent cysteine transport and glutathione formation (Song et al., 2018). SLC7A11 is also a target of p53^3KR^, and p53^3KR^ transcriptionally downregulates SLC7A11 expression and induces ferroptosis upon cysteine uptake limitation and ROS induction (Jiang et al., 2015). Moreover, transcription factor Nrf2 upregulates system Xc^-^ in various cancers, and this increases the redox-sensitivity of the cell and induces ferroptosis (Sun X. et al., 2016).

### Inactivation of GPX4 Induces Ferroptosis

Elucidating the mechanism of RSL3-mediated cell death provided further major insight into the regulation of ferroptosis. Cell death induced by erastin and RSL3 share common features of ferroptosis, such as a dependence on iron and ROS; however, RSL3 toxicity is dependent on either the voltage-dependent anion-selective channel protein 2/3 (VDAC2/VDAC3) (Yagoda et al., 2007) or system X_c_^-^ indicating that it functions through a different initiating mechanism (Yang et al., 2014). Analysis of affinity-based chemoproteomics with LC-MS and western blotting confirmed the interaction between GPX4 and RSL3 (Yang et al., 2014), and further study found that RSL3 covalently interacts with the selenocysteine in the active site of GPX4 to inhibit its enzymatic activity (Yang et al., 2016). Knockdown of GPX4 by shRNA induces ferroptosis, while overexpression of GPX4 renders cells resistant to RSL3 toxicity (Yang et al., 2014), and this suggests that different initiating mechanisms converge to a similar form of ferroptotic cell death. GPX4, which uses GSH as an essential cofactor, catalyzes the reduction of hydrogen peroxide and organic hydroperoxides, especially lipid-hydroperoxides, to water and the corresponding alcohols, respectively (Figure 1).

### Role of ROS and Lipid Peroxidation in Ferroptosis

Erastin induces ferroptosis by inhibiting system X_c_^-^ and thus inducing the accumulation of ROS, which have been shown to be lipid ROS according to flow cytometry assays using the fluorescent probes dichlorofluorescein (DCF), H2DCFDA, and C11-BODIPY (Yagoda et al., 2007; Dixon et al., 2012). Further support for the involvement of lipid ROS in ferroptosis comes from a study showing that RSL3 induces ferroptosis by binding to and inhibiting GPX4, thereby increasing lipid peroxidation as measured by C11-BODIPY fluorescence (Yang et al., 2014).

Several lipophilic antioxidants have been identified as strong suppressors of erastin-induced cell death, including α-tocopherol, butylated hydroxytoluene, β-carotene, ferrostatin-1 (Fer-1), and liproxstatin-1. Fer-1 and liproxstatin-1 are regarded as specific ferroptosis suppressors because they suppress the cell death induced by ferroptosis inducers (erastin, RSL3) and fail to save cells from apoptosis or necroptosis induced by staurosporine and H_2_O_2_, respectively (Dixon et al., 2012; Yang et al., 2014).

Lipidomics analysis revealed that polyunsaturated fatty acids (PUFAs) are the most susceptible lipids to peroxidation in the course of ferroptosis compared to other classes of lipids (Yang et al., 2016). A recent study identified acyl-CoA synthetase long-chain family member 4 (ACSL4) as an essential component of ferroptosis through a genome-wide CRISPR-based genetic screen and a microarray analysis of ferroptosis-resistant cell lines. ACSL4-enriched cellular membranes with long polyunsaturated omega-6 fatty acids and *GPX4*^-^/*ACSL4*^-^ double-knockout cells show marked resistance to ferroptosis (Doll et al., 2017). Mechanistically, ACSL4 is required for activation of PUFAs, especially arachidonic acid (AA) and adrenic acid (AdA) to AA-CoA and AdA acyl co-A derivatives (Hirschhorn and Stockwell, 2018). Next, lysophosphatidylcholine acyltransferase 3 (LPCAT3) can esterify these derivatives into PE (phosphatidylethanolamine) to form AA-PE and AdA-PE, which then be catalyzed by an iron-containing enzyme lipoxygenase (LOX) to generate fatty acid hydroperoxides in a stereospecific manner (Stockwell et al., 2017; Hirschhorn and Stockwell, 2018) (Figure 1). Lipid peroxidation seems play a role in the final stage of ferroptosis, and this is evidenced by pronounced upregulation of aldo-keto reductase family 1 member C family genes in cell lines that are resistant to erastin-induced ferroptosis (Dixon et al., 2014; Stockwell et al., 2017). The products of these genes can detoxify the end products of oxidized PUFAs, such as 4-hydroxynonenal which are likely produced by the oxidative lipid fragmentation processes that occur during the execution of ferroptosis (Stockwell et al., 2017).

### The Role of Iron in Ferroptosis

Although the exact role of iron in ferroptosis remains enigmatic, there is considerable evidence that iron is a necessary component in this form of oxidative cell death (Dixon et al., 2012; Li et al., 2017). It has been shown that erastin induces ferroptosis through the accumulation of ROS in the cytosol as measured by flow cytometry using the fluorescent probe DCF when incubation with exogenous sources of iron rather than other divalent transition metal ions (Cu^2+^, Mn^2+^, Ni^2+^, and Co^2+^), and this process is suppressed by co-treatment with the iron chelator DFO (Dixon et al., 2012). Therefore, the state of cellular free iron seems to be important to ferroptosis induction. Consistently, studies have demonstrated that proteins that control cellular iron balance, such as transferrin, ferritin, ferroportin, and other iron-containing proteins for iron uptake, storage, utilization, and degradation participate in promoting ferroptosis. In fact, treatment with recombinant iron-loaded rather than iron-free transferrin promoted ferroptotic cell death (Gao et al., 2015). Moreover, iron chelation, depletion of transferrin from serum, and knockdown of TfR1 prevents erastin-induced ferroptosis. Erastin-induced cell death can also be inhibited by Fer-1, a specific ferroptosis inhibitor (Dixon et al., 2012), which also blocks cell death triggered by either full amino acid starvation or cystine starvation (Gao et al., 2015). Inhibition of ferritin autophagy, referred to as ferritinophagy, through blockage of autophagy or knockdown of cargo receptor NCOA4 (nuclear receptor coactivator 4) which recruits ferritin to autophagosomes for lysosomal degradation, abrogates the accumulation of labile iron and ROS, and prevents ferroptosis (Santana-Codina and Mancias, 2018) (Figure 1). In addition, iron response element binding protein 2 was identified as an essential gene for the induction of ferroptosis due to its role in the regulation of iron metabolism and iron accumulation (Dixon et al., 2012). Collectively, these results strengthen the view that iron is necessary in initiating ferroptosis.

Although iron is necessary for ferroptosis, it remains unclear how iron acts in ferroptosis. One hypothesis is that iron acts as free iron and generates hydroxyl radicals or hydroperoxyl radicals through the Fenton reaction, which is an important source of ROS (Dixon and Stockwell, 2014). Another hypothesis is that iron functions in ferroptosis as a cofactor of iron-containing enzymes. As mentioned before, lipoxygenases are a family of iron-containing enzymes that mediate PUFA oxidation and exert a lethal effect under conditions of GSH depletion (Yang et al., 2016). Other studies have found that other iron-containing enzymes – the hypoxia-inducible factor prolyl hydroxylases (HIF-PHDs) – are related to the process of ferroptosis (Speer et al., 2013; Karuppagounder et al., 2016) (Figure 1). Also, adaptaquin, a specific inhibitor of HIF-PHD enzymes, reduced neuronal death and behavioral deficits after intracranial hemorrhage (ICH) in a rodent model without affecting total iron or zinc distribution in the brain (Karuppagounder et al., 2016). The protection from oxidative death *in vitro* or from ICH *in vivo* by adaptaquin was associated with suppression of the activity of activating transcription factor 4 (ATF4) rather than activation of a HIF-dependent pro-survival pathway.

### Other Molecules Regulate Ferroptosis

Several other metabolic pathways and molecules regulate ferroptosis sensitivity. As a limited building block of GSH, the level of cysteine acts as the upstream signal of ferroptosis. Cysteine starvation and inhibition of system X_c_^-^-induced ferroptosis can be rescued by the trans-sulfuration pathway (biosynthesis of cysteine from methionine) in some cells. Cysteinyl-tRNA synthetase (CARS) was recently discovered to be involved in this pathway, and knockdown of CARS increases intracellular free cysteine and inhibits erastin-induced ferroptosis (Hayano et al., 2016; Stockwell et al., 2017). However, cysteine deficiency does not induce the generation of lipid peroxidation and ferroptosis when there is a lack of glutamine or when there is inhibition of glutaminolysis (Gao et al., 2015; Stockwell et al., 2017). Glutamine is a major cellular energy source and can provide elements for biosynthesizing amino acids, nucleic acids, and lipids by generating intermediates through glutaminolysis. Glutaminase 1 (GLS1) and glutaminase 2 (GLS2) both catalyze glutamine into glutamate as the first reaction of glutaminolysis, but only suppression of GLS2 prevents ferroptosis, which is transcriptionally controlled by the P53 P47S variant (Jennis et al., 2016). Mevalonate-derived antioxidant coenzyme Q10 (CoQ10), which is derived from the mevalonate pathway, is a negative regulator of ferroptosis by reducing the accumulation of lethal lipid peroxidation induced by FIN56 (Shimada et al., 2016) (Figure 1). Many other molecules and metabolic pathways need to be explored.

## Neonatal Brain Injury

Neonatal brain injury is a major public health issue and is a leading cause of neonatal mortality and morbidity, especially in preterm infants. Neonatal brain injury is not a single well-defined entity, and many factors contribute to such injury, but the most common etiologies are hypoxic–ischemic encephalopathy in term infants and intraventricular/periventricular hemorrhage in preterm infants (Gale et al., 2018). Brain injury evolves over time and goes through different stages, and multiple mechanisms contribute to this process, including energy depletion, excitatory amino acids, mitochondrial impairment, generation of ROS, and inflammation, all of which lead to different types of cell death (Hagberg et al., 2014; Sun et al., 2017; Albertsson et al., 2018; Davidson et al., 2018; Nazmi et al., 2018). Apoptosis and necrosis have been identified as the two main mechanisms of cell death in many different variants of brain injury (Li et al., 2010; Zhu et al., 2010; Northington et al., 2011; Thornton et al., 2017), but more and more studies have demonstrated that different forms of cell death occur simultaneously or successively (Sun Y. et al., 2016; Xie C. et al., 2016; Sun et al., 2017). After the discovery of ferroptosis, recent studies have also demonstrated connections between ferroptosis and neurological diseases (Tonnus and Linkermann, 2016; Hambright et al., 2017; Zille et al., 2017).

Compared to the adult brain, the neonatal brain has a high rate of oxygen consumption, high concentrations of unsaturated fatty acids, and low concentrations of antioxidants, which make it particularly sensitive to oxidative damage (Blomgren et al., 2003). The PUFA content of the brain increases during gestation and indicates that the preterm brain is even more sensitive to lipid peroxidation than the term brain and that lipid peroxidation might be a major factor in the white-matter damage seen in preterm infants who suffer from brain injury (Millar et al., 2017). Furthermore, the brain’s endogenous antioxidant defense mechanisms show less activity in the immature brain compared to the mature brain (Lafemina et al., 2006). Altogether, this suggests that the immature brain is more sensitive to oxidative stress-induced cell death and brain injury. Because perinatal hypoxia and ICH are two dominant causes of neonatal brain injury, we focus on the potential contribution of ferroptosis on asphyxia and ICH-induced neonatal brain injury.

### Ferroptosis and Peripartum Asphyxia

Despite important progress in obstetric and neonatal care in recent years, perinatal asphyxia is still one of the leading causes of death and adverse developmental outcomes (Zhu et al., 2009; Azzopardi et al., 2016; Liu et al., 2016). Perinatal hypoxic-ischemic insult-induced cell death peaks at 24–48 h, but this pathological process continues for weeks after injury (Geddes et al., 2001; Fleiss and Gressens, 2012), and this long-lasting cell death causes significant loss of brain volume as indicated by cerebral MRI (Wideroe et al., 2011). Further studies that delayed the chronic phase of cell death for several days after acute injury reduced brain injury and tissue loss volume (Han et al., 2014; Xie et al., 2014). Several types of cell death have been shown to be involved in neonatal brain injury. Apoptosis, necrosis, and autophagy are the three types of cell death that have been commonly identified (Zhu et al., 2005; Li et al., 2010; Xie C. et al., 2016), but with the identification of ferroptosis, new potential mechanisms of cell death should be taken into consideration.

Excitotoxicity, oxidative stress, and inflammation play important roles in the mechanism of neonatal hypoxic-ischemic (HI) brain injury (Li et al., 2011; Bi et al., 2015). HI results in depolarization of neurons and glial cells and the subsequent release of excitatory amino acids such as glutamate into the extracellular space. Elevated glutamate has been documented in the cerebrospinal fluid of infants who have suffered severe HI injury (Pu et al., 2008). Accumulated glutamate activates the NMDA receptors that mediate normal brain development and function by promoting the proliferation and migration of neuronal precursors, synaptic development, and plasticity (Rocha-Ferreira and Hristova, 2015), but excessive glutamate is also a risk factor for triggering ferroptosis (Figure 1). Studies have shown that GSH is reduced after hypoxia in the neonatal periventricular white matter and in primary cultured oligodendrocytes when exposed to hypoxia or to conditioned medium from hypoxic microglial cells (Kaur et al., 2010). Such glutamate toxicity-mediated X_c_^-^ inhibition and GSH depletion is also established in a model system of oxytosis in immortalized hippocampal neurons, the HT22 cell line, which is widely used to study mechanisms of oxidative cell death involving enhanced lipid peroxidation. As mentioned before, recent data demonstrated that glutamate-mediated X_c_^-^ inhibition in oxytosis resembles the major features of ferroptosis. Thus, glutamate toxicity *in vivo* might also involve ferroptosis mechanisms similar to those described for erastin and RSL3 in cancer cells.

Iron is another dangerous factor during HI injuries. Iron homeostasis in the brain involves the regulation of iron movement between the blood and the brain, between the intracellular and extracellular spaces in the brain, and between different iron pools within such spaces (Ke and Qian, 2007; Garton et al., 2016). This homeostasis is maintained by a series of iron transport proteins (e.g., transferrin) and iron storage proteins (e.g., ferritin). Under normal physiological conditions, most of the brain iron is sequestered within storage proteins because protein-bound iron is safe while free iron can generate highly damaging reactive molecules, which can cause damage to proteins and nucleotides and lipid peroxidation via Fenton chemistry. During the actual HI event, protein-bound iron is liberated from its binding proteins due to the low intracellular pH. After HI, the rapid accumulation of iron is seen in damaged neurons and in the periventricular white matter of neonatal rats (Rathnasamy et al., 2011), and increased levels of iron-bound proteins IRP1, IRP2, and TfR, and accumulated free iron are seen in the serum and cerebrospinal fluid of human infants (Shouman et al., 2008). Intracerebral injection of the lipid-soluble form of transferrin (apotransferrin) attenuates white matter damage in a neonatal rat model of cerebral HI, which suggests that overloaded free iron can protect cells from oxidative stress (Guardia Clausi et al., 2012). Indeed, after HI, production of free radicals is activated, which then attack unsaturated fatty acids and lead to the production of neurotoxic lipid peroxides (LPOs). Rodent models have shown abundant formation of LPOs in the brain after HI, and the serum levels of malondialdehyde, which is an end-product of lipid peroxidation, increase in neonates after HI (El Bana et al., 2016). Serum LPO concentrations increase significantly in asphyxiated infants, and this correlates with the clinical grading of hypoxic–ischemic encephalopathy and mortality (Ramy et al., 2016). The level of LPO, as well as the severity of cell damage, can be reduced by the iron chelator DFO (Peeters-Scholte et al., 2003; Rathnasamy et al., 2011).

Ferroptosis has been reported in ischemic injuries in other tissues *in vivo* and *in vitro*, and different iron chelators and their analogs have shown great potential in preventing these injuries. Treatment with DFO reduces the infarct size in hearts suffering from ischemia/reperfusion stress (Gao et al., 2015) and protects against acute renal failure and organ damage in a model of severe kidney ischemia/reperfusion injury (Linkermann et al., 2014). A study in a newborn mouse model found that iron pretreatment aggravates periventricular cystic white-matter lesions (Dommergues et al., 1998), and this has been proposed to be related to iron overload-induced oxidative stress (Gong et al., 2016). Erythropoietin (EPO) is thought to have neuroprotective effects through multiple mechanisms, including neurotrophic, anti-oxidant, anti-apoptotic, and anti-inflammation activities and the promotion of neural stem cell proliferation and differentiation (Wang et al., 2004; Juul and Pet, 2015). Furthermore, EPO promotes erythropoiesis, which increases iron utilization and potentially reduces free iron (Bany-Mohammed et al., 1996). Preterm infants who received EPO treatment had significantly reduced serum ferritin accompanied by reduced serum lipid peroxidation (Akisu et al., 2001), and our clinical studies have shown that EPO treatment reduces neurological sequelae in both term and preterm infants (Zhu et al., 2009; Song et al., 2016). During a hypoxic insult, HIF is stabilized by PHDs and translocates into the nucleus where it turns on transcription of EPO. Previous studies have shown that EPO stimulates the activity of GPX4 and inhibits lipid peroxides and thus might act as an inhibitor of ferroptosis (Genc et al., 2002) (Figure 1). The neuroprotective effect of EPO might be related to reduced levels of unbound iron and oxidative stress and thus to reduced levels of ferroptosis (Bailey et al., 2014).

### Ferroptosis and Intracranial Hemorrhage in Preterm Infants

Intracerebral hemorrhage is one of the most common complications in preterm infants, and its rate of occurrence increases with decreasing birth weight and decreasing gestational age (Bolisetty et al., 2014). ICH occurs in 15% of very preterm infants, and more than half of all infants with severe ICH develop post-hemorrhagic ventricular dilation and around 30% develop cerebral palsy (Bolisetty et al., 2014). Currently there is no efficient therapy to protect infants from neurological disability as a result of ICH. ICH-induced brain injury includes both the primary injury, caused by the physical presence of a hematoma, and secondary injury caused by the effect of neurotoxic compounds released from the hematoma, including free iron and cell-free hemoglobin, and iron accumulation contributes to ventricular dilation (Chen et al., 2015; Xiong et al., 2016; Garton et al., 2017). It has also been shown that iron chelator treatment alleviates ventricular dilation after ICH, and this confirms the important role of iron in the development and progression of brain injury after ICH (Meng et al., 2015).

There is growing evidence that clot-derived factors such as hemoglobin, iron, and fibrinogen have an important role in ICH-induced secondary injury (Ley et al., 2016; Garton et al., 2017; Petersen et al., 2018). Hemoglobin and iron-induced neuronal toxicity have attracted much attention by neonatologists (Li et al., 2017), and hemoglobin and its metabolites are cytotoxic and are capable of inducing oxidative stress and inflammation (Agyemang et al., 2017). Hemoglobin is degraded in the brain by heme oxygenase into iron, carbon monoxide, and biliverdin, and a large amount of iron is released from hemoglobin into the extracellular space following hemorrhage (Savman et al., 2001), and this can result in free radical production through the Fenton reaction and ultimately cause oxidative damage to DNA, proteins, and lipids (Figure 1). Non-protein-bound iron is elevated in cerebrospinal fluid from preterm infants with post-hemorrhagic ventricular dilatation (Savman et al., 2001). The infusion of hemoglobin and its degradation products to rats causes ventricular dilation and brain damage (Hua et al., 2003) and leads to upregulation of heme oxygenase-1 and ferritin and to increased iron deposition (Strahle et al., 2014). Treatment with DFO, an iron chelator, has shown promise as a treatment for ICH due to its beneficial effects in reducing iron-induced neuronal death and inflammation (Hatakeyama et al., 2013; Strahle et al., 2014; Karuppagounder et al., 2018).

Although studies have shown that necrosis, apoptosis, and autophagy contribute to brain injury after ICH (Chen et al., 2012; Lule et al., 2017; Li et al., 2018), more and more evidence indicates that cell death occurs through other mechanisms as well (Zille et al., 2017; Li et al., 2018). Iron toxicity and glutamate accumulation, and thus ROS generation, also occur after ICH, which supports the hypothesis that ferroptosis is involved in these brain injuries (Dixon et al., 2014), and it has been shown that experimental ICH exhibits many features of ferroptotic and necroptotic cell death (Zille et al., 2017). In addition, ultrastructural analysis of neuronal death after ICH has shown the co-occurrence of ferroptosis, autophagy, and necrosis (Li et al., 2018). Fer-1, a specific inhibitor of ferroptosis, prevents neuronal death and reduces iron deposition induced by hemoglobin in organotypic hippocampal slice cultures, and it also improves neurological function in mice after ICH. Fer-1 also reduces lipid ROS production and attenuates the increased expression level of PTGS2 and its gene product cyclooxygenase-2 both *ex vivo* and *in vivo* (Li et al., 2017). All of these adult animal model studies confirm that ferroptosis occurs in both the collagenase-induced ICH model and in autologous blood injection-induced cell death (Li et al., 2017; Zhang et al., 2018). However, there is no report yet regarding ferroptosis in neonatal intraventricular hemorrhage. An intervention study using DFO in a neonatal rat germinal matrix hemorrhage model showed neuroprotection in terms of both brain morphology and behavior (Klebe et al., 2014), and this indicates that ferroptosis might play an even more important role in the secondary injury after neonatal ICH. This requires further investigation to provide evidence for potentially clinically translatable therapeutic strategies.

## Concluding Remarks

As a novel form of regulated cell death, ferroptosis occurs in cells when iron accumulation and lipid peroxidation are activated. However, our understanding of ferroptosis is still at an early stage, and no specific biomarkers have been found for the identification of ferroptosis *in vivo*. Overall, ferroptosis is largely defined through pharmacological activators and inhibitors. According to pharmacological effects of pro- and anti-ferroptotic substances, the mechanism of ferroptosis mainly includes iron toxicity, the inactivation of GPX4 and system X_c_^-^, and ultimately lipid peroxidation. Ferroptosis is involved in adult ischemic and intraventricular hemorrhage-induced neuronal cell death, and ferroptosis inhibition reduces neuronal death and behavioral deficits. Even though there are so far no direct reports of ferroptosis in neonatal brain injury, there is evidence to suggest that ferroptosis should be more prone to occur in the neonatal brain. To mimic PVL *in vitro*, cultured oligodendrocytes under cystine-free conditions showed depleted GSH and cell death, and this type of cell death could be blocked by ferroptosis-inhibiting ferrostatins (Skouta et al., 2014). Considering the emerging evidence, ferroptosis should be investigated further in models of neonatal brain injury and should be considered as a potential therapeutic target for the treatment of neonatal brain injury.

## Author Contributions

CZ conceptualized and designed the review, and participated in literature searching and analysis, prepared the initial draft, and finalizing the manuscript. YNW participated in literature searching and collection and analysis, and participated in drafting and finalizing the manuscript. JS, YFW, XW, and CC participated in drafting and finalizing the manuscript.

## Conflict of Interest Statement

The authors declare that the research was conducted in the absence of any commercial or financial relationships that could be construed as a potential conflict of interest.
